# Influence of servant leadership on the life satisfaction of basic education teachers: the mediating role of satisfaction with job resources

**DOI:** 10.3389/fpsyg.2023.1167074

**Published:** 2023-10-30

**Authors:** Ronald Quinteros-Durand, Robinson B. Almanza-Cabe, Wilter C. Morales-García, Oscar Mamani-Benito, Liset Z. Sairitupa-Sanchez, Lucy Puño-Quispe, Jacksaint Saintila, Renán Saavedra-Sandoval, Alcides Flores Paredes, Andrés Alexis Ramírez-Coronel

**Affiliations:** ^1^Unidad de Posgrado en Educación, Escuela de Posgrado, Universidad Peruana Unión, Lima, Peru; ^2^Escuela Profesional Gestión Pública y Desarrollo Social, Universidad Nacional de Moquegua, Moquegua, Peru; ^3^Escuela de Medicina Humana, Facultad de Ciencias de la Salud, Universidad Peruana Unión, Lima, Peru; ^4^Unidad de Posgrado en Salud, Escuela de Posgrado, Universidad Peruana Unión, Lima, Peru; ^5^Facultad de Derecho y Humanidades, Universidad Señor de Sipán, Chiclayo, Peru; ^6^Escuela Profesional de Psicología, Facultad de Ciencias de la Salud, Universidad Peruana Unión, Lima, Peru; ^7^Escuela Profesional de Psicología, Facultad de Ciencias de la Salud, Universidad Peruana Unión, Juliaca, Peru; ^8^Escuela de Medicina Humana, Universidad Señor de Sipán, Chiclayo, Peru; ^9^Escuela de Posgrado, Universidad César Vallejo, Tarapoto, Peru; ^10^Escuela Profesional de Educación Física, Universidad Nacional del Altiplano, Puno, Peru; ^11^Nursing Career, Azogues Campus, Catholic University of Cuenca, Cañar, Ecuador; ^12^Laboratory of Psychometry, Comparative Psychology and Ethology, Catholic University of Cuenca, Cuenca, Ecuador; ^13^Health and Behavior Research Group (HBR), Catholic University of Cuenca, Cuenca, Ecuador

**Keywords:** servant leadership, life satisfaction, resources, education, teachers

## Abstract

**Background:**

Life satisfaction is essential for teachers’ work performance and student learning. Additionally, servant leadership has been shown to be one of the best leadership practices as it promotes employee well-being and satisfaction. Moreover, satisfaction with job resources acts as a mediator in the relationship between servant leadership and life satisfaction by influencing individual and collective performance in the organization.

**Objective:**

This research aimed to evaluate the mediating role of satisfaction with job resources in the relationship between servant leadership and life satisfaction.

**Methods:**

The study was cross-sectional and explanatory. 620 teachers aged between 20 and 62 years (*M* = 35 and SD = 9.49) participated in the study. Structural Equation Modeling (SEM) was used to measure life satisfaction, service leadership, and job resource satisfaction through the use of questionnaires.

**Results:**

The results indicated that the model obtained an adequate fit, χ^2^ = 2,658, df = 551, *p* < 0.001, CFI = 0.941, TLI = 0.936, RMSEA = 0.079, SRMR = 0.070. The results confirm the positive influence of leadership on satisfaction with resources and life satisfaction. Additionally, a positive influence of satisfaction with job resources on life satisfaction was observed. Moreover, the mediation of job resources in servant leadership and life satisfaction was confirmed.

**Conclusion:**

Servant leadership, supported by satisfaction with job resources, can reduce effort and associated costs, stimulate personal growth and learning, and improve the well-being of teachers.

## 1. Introduction

Life satisfaction is a critical factor for both job performance and student learning. Teachers who experience a high level of life satisfaction are more motivated and committed to their work, resulting in better performance in the classroom (Nagarajan et al., 2022). This also enables greater empathy, control of their classes, and the creation of a positive learning environment, improving the relationship between teachers and their students (Guess and McCane-Bowling, 2016; Nie et al., 2019; Braun et al., 2020). As a result, teachers can better resolve conflicts and deal with stressful situations in the classroom (Moore, 2008). Additionally, they are less likely to suffer from mental and physical health problems, allowing them to stay healthy and energized to fulfill their daily tasks (Randall et al., 2021). This helps them to achieve a better balance between their work and personal life (Carroll et al., 2021).

Well-being is defined as a combination of feeling good and functioning well. It includes positive emotional experiences, potential development, autonomy, purpose, and healthy relationships (Huppert, 2009). It is a sustainable state that promotes personal and collective development and prosperity (Holck et al., 2001). Well-being is associated with success in various areas, such as work, personal and interpersonal, and is related to positive outcomes in health, life satisfaction, and economic performance (Oishi et al., 2007; Diener, 2012). Furthermore, it improves academic performance (Crippen and Willows, 2019; Shen et al., 2020). Life satisfaction is associated with leadership, as an organization that practices service-oriented leadership will have an improved perception of satisfaction (Kroumova’s, 2020). Therefore, it is recommended that leaders adopt a service-oriented style and that organizations hire personnel with these qualities (Li et al., 2018). Leader-member Exchange (LMX) focuses on the relationship between leaders and followers and their influence on job satisfaction. Employees with high-quality LMX have greater information exchange with their leaders, as well as higher levels of trust and support (Tu and Lu, 2016; Fu et al., 2019; Pan et al., 2021). Due to its people-centered approach, followers are expected to have greater well-being and satisfaction, making service-oriented leadership a significant factor for individual life satisfaction (Van Dierendonck et al., 2014; Li et al., 2018; Latif et al., 2022).

In the educational field, several studies emphasize the importance of exercising servant leadership by teachers (Poobalan and Talip, 2020). This is one of the best leadership practices for addressing unexpected challenges, such as the coronavirus pandemic, as it focuses on empowerment, engagement, and collaboration (Eva et al., 2019). Servant leadership is based on a holistic approach encompassing ethical, rational, emotional, relational, and spiritual dimensions (Sendjaya and Sarros, 2002). Therefore, it is a people-oriented leadership approach that prioritizes the needs and interests of subordinates and redirects personal care toward them in organizations and communities. This creates a positive work environment that increases subordinates’ commitment to their roles and organizations (Newman et al., 2017; Eva et al., 2019). Additionally, servant leadership helps improve teacher professionalism (Poobalan and Talip, 2020). These leaders see their subordinates as their top priorities and work for their benefit, creating an environment in which they foster positive relationships, provide empowerment, help them grow and succeed, act ethically, and possess cognitive skills, generating value outside the organization (Greenleaf, 1977b).

Servant leadership has significantly impacted employees’ life satisfaction, as servant leaders focus on surpassing their own interests and on the growth and development of their environment. These actions allow them to achieve professional goals and perform at their best, which in turn makes them happier and more satisfied with their lives (Chughtai, 2018). Several previous studies used different variables as mediators to better explain the mechanism by which servant leadership influences followers’ outcomes (Chughtai, 2019; Latif et al., 2021; Dami et al., 2022b). For example, life goals and psychological needs acted as mediators in the relationship between servant leadership and teachers’ life satisfaction (Liaqat et al., 2022). Furthermore, job satisfaction has been shown to be a significant mediator between service leadership and life satisfaction at the higher education level (Latif et al., 2021). The mediating role of satisfaction of autonomy, competence, and relatedness needs at work is also discussed (Chiniara and Bentein, 2016). Organizational-level studies suggest that there are characteristics of the work environment that influence workers’ perception of satisfaction (Lee and Park, 2021). These characteristics relate to the job resources that workers have in an organization. According to the Job Demands-Resources (JD-R) theory (Demerouti et al., 2001; Bakker and Demerouti, 2018), satisfaction with job resources encompasses people’s well-being in relation to different factors, which favor individual and collective performance. These factors or resources can be found at different levels: leader, considering the boss-supervisor relationship, clarity of instructions, feedback, and recognition for achievements; tasks, which involve the job characteristics, availability of time and materials to meet objectives; team, which involves the relationship with co-workers in terms of cooperation, coordination, efficiency, and creativity; and finally, organization, which refers to job conditions such as salary, reward system, benefits, development opportunities, training, and learning (Chughtai, 2018; Spontón et al., 2019).

### 1.1. Theoretical framework

#### 1.1.1. Constructive development theory

Kegan’s theory of constructive development focuses on how individuals construct meaning and epistemological knowledge throughout different stages of development. Each stage represents a set of common organizational principles used to construct experiences, not differing by the content of experiences or how things are done, but rather by the principles by which thinking, feeling, and social relationships are constructed and organized. The transition from one stage to another involves a change in subject and object and represents an increase in the responsibility individuals have over the meaning they construct. There are six stages or “equilibria” of development that affect both emotional and relational function (incorporative, impulsive, imperial, interpersonal, institutional, and interindividual). To support individuals in their transition toward more complex stages, an environment that attends to the dominant stage of the individual and challenges them to access the next stage is necessary. Professionals working with individuals at different stages must adapt their approaches to adequately support and challenge their clients in their path toward psychological maturity. Teachers can build a supportive and challenging environment for interindividuals to help promote their ongoing growth and development (Eriksen, 2006).

#### 1.1.2. Maslow’s hierarchy of needs

Maslow’s Hierarchy of Needs (HON) provides a framework for understanding human motivation that has been widely applied in the educational context. This theory suggests that individuals move through a series of hierarchical motivations, starting with physiological needs, safety, love, and belongingness, before moving on to higher-order needs such as self-esteem, self-actualization, and self-transcendence. According to Maslow, if lower-level needs are not met, individuals will experience negative physiological and psychological consequences. In the educational context, this implies that students’ basic needs must be met before they can engage in higher-level thinking and learning. Therefore, the HON can inform teaching strategies and approaches to support students’ physiological and psychological well-being, allowing them to reach their full potential (Maslow, 1954; Soni, 2016).

#### 1.1.3. Motivation-hygiene theory

The Motivation-Hygiene Theory, developed by Frederick Herzberg, has been proposed as an alternative method for measuring job satisfaction and its determinants. This theory suggests that satisfaction and dissatisfaction are completely separate issues, and there are two factors: motivator or satisfier factors and hygiene or dissatisfier factors. Motivator factors are job factors that enhance satisfaction or motivation, such as the work itself, responsibility, recognition, achievement, and growth. In contrast, hygiene factors, such as company policy, salary, working conditions, and supervision, decrease dissatisfaction. Both satisfaction and dissatisfaction at work are crucial for organizations to manage because they ultimately impact productivity and effectiveness (Herzberg et al., 1959; Herzberg and Hamlin, 1961; Triandis and Herzberg, 1967). For this reason, it is essential that organizations understand the factors that create satisfaction and contribute to improved morale, which in turn will bring greater happiness and self-fulfillment. The discussion about job satisfaction and dissatisfaction is mainly developed from Herzberg’s theory, and employee morale is a crucial factor associated with their motivation, reflecting their perception regarding their job, managers, and the organization itself (Brenner et al., 1971; Sachau, 2007). The role of teachers in achieving educational goals and objectives cannot be ignored. Therefore, it is important to improve employees’ effectiveness and understand the factors that can create satisfaction, as satisfied employees can contribute to improved morale and productivity.

#### 1.1.4. Job characteristics model

The Job Characteristics Model (JCM) suggests that enriched or complex jobs lead to higher job satisfaction, motivation, and job performance (Hackman and Oldham, 1975, 1976, 1980). The model postulates that five core job characteristics: skill variety, task identity, task significance, autonomy, and feedback, influence three critical psychological states: experienced meaningfulness of the work, experienced responsibility for the outcomes of the work, and knowledge of the actual results of the work activities. These psychological states, in turn, affect job outcomes such as internal work motivation, growth satisfaction, general job satisfaction, job effectiveness, and absenteeism. Hackman and Oldham also proposed three moderators of the relationships between job characteristics, psychological states, and job outcomes: growth need strength, knowledge and skill, and context satisfaction. They also emphasized the importance of task identity, autonomy, and feedback in fostering motivation, learning, and skill development for teachers (Coelho and Augusto, 2010). Thus, the JCM provides a useful framework for understanding how job design can influence teacher motivation and performance.

#### 1.1.5. The dispositional approach

The dispositional approach involves the measurement of personal characteristics and the assumption that such measures can aid in explaining individual attitudes and behavior (Funder and Ozer, 1983). Although distinctions are sometimes made between the concepts of personal dispositions, traits, personality, and individual characteristics, these terms are used almost interchangeably in the literature (Bem and Allen, 1974), Each of these terms is based on a set of common assumptions: that it is possible to characterize people on certain dimensions, that these dimensions have some stability over time, and that these dimensions are useful in predicting individual behavior across situations (Staw and Ross, 1985).

#### 1.1.6. The job demands-resources (JD-R) theory

The Job Demands-Resources (JD-R) Theory (Demerouti et al., 2001; Chughtai, 2016) provides a useful framework to examine how satisfaction with job resources influences the relationship between service leadership and life satisfaction among teachers. This is because the JD-R theory argues that job demands, and job resources influence employees’ well-being and performance. Job demands are physical, psychological, social, or organizational aspects that require sustained effort and may lead to exhaustion and stress (Demerouti et al., 2001). On the other hand, job resources are aspects that help employees cope with job demands, achieve their goals, and develop personally and professionally (Bakker and Demerouti, 2018).

In the educational context, service leadership can be considered as a job resource that influences teachers’ life satisfaction. Servant leaders, by focusing on the well-being and development of their subordinates, can provide a work environment where teachers’ needs are met, and job demands are reduced (Greenleaf, 1977a; Van Dierendonck et al., 2014). Moreover, by providing emotional, instrumental, and social support to teachers, servant leaders can improve satisfaction with job resources, and ultimately, teachers’ life satisfaction (Chughtai, 2018; Eva et al., 2019).

The JD-R theory suggests that job resources act as mediators in the relationship between job demands and employees’ well-being and performance outcomes (Bakker and Demerouti, 2017). In this sense, satisfaction with job resources can play a mediating role in the relationship between service leadership and teachers’ life satisfaction. For instance, the support and guidance provided by servant leaders can enhance empowerment and satisfaction with job resources related to leadership, tasks, team, and organization (Lin et al., 2020). In turn, higher satisfaction with these job resources can lead to higher satisfaction with teachers’ life (Kaur, 2018; Marcionetti and Castelli, 2022; Dami et al., 2022a).

In this sense, it is important to explore how different job resources, such as leader support, quality of relationships with colleagues, access to materials and development opportunities, and job conditions, can affect the relationship between service leadership and teachers’ life satisfaction. This will allow a better understanding of the underlying mechanisms in this relationship and provide valuable information for interventions and policies aimed at improving life satisfaction and well-being of teachers in specific educational contexts such as Peru.

### 1.2. Literature review

#### 1.2.1. Life satisfaction

Life satisfaction plays a fundamental role in subjective well-being, referring to how individuals evaluate their life and experience their emotions (Diener, 1984; Diener et al., 1999). Life satisfaction is a crucial aspect of human life, as it is considered an indicator of an individual’s overall well-being. It is a cognitive measure that addresses a person’s evaluation of their life in terms of achievements and expectations (Pavot and Diener, 1993). Placing greater emphasis on life satisfaction means paying special attention to how an individual’s perception of their overall life influences their emotional and psychological well-being. High levels of life satisfaction are associated with various positive outcomes such as better job performance, stronger interpersonal relationships, greater resilience in difficult situations, and better physical and mental health (Ferguson, 2011; Diener, 2013). This variable is defined as a cognitive process where individuals evaluate the quality of their lives based on their own criteria, comparing their standards with their current condition (Calderón-De la Cruz et al., 2018). Therefore, it is an essential indicator of people’s subjective well-being (Schnettler et al., 2014). Studies indicate that it is related to having a better quality of life (Dolan et al., 2013), a better perception of family satisfaction (Ni et al., 2021), better interpersonal relationships (Ruvalcaba-Romero et al., 2017), and a better economic situation (Mikucka et al., 2017). However, the socioeconomic level influences the relationship between income or resources and individual well-being (Salinas-Jiménez et al., 2010), and being more negative in developing countries (Tasiemski et al., 2021).

#### 1.2.2. Servant leadership

Servant leadership refers to a leader’s tendency to guide and motivate followers to provide hope and establish quality relationships (Greenleaf, 1977a). This begins when someone adopts a position of servant to promote growth in others (Mittal and Dorfman, 2012). In this way, leadership is authentic when there is a desire to help others and not to benefit oneself or exert power (Greenleaf, 1977b; Johnson, 1997). Therefore, the primary goal of a servant leader is to ensure that subordinates develop and grow into servant leaders (Amah, 2018). This leadership style is considered appropriate for the educational environment, as leadership in this context requires specific skills and abilities (Latif et al., 2021). Scientific literature has confirmed the positive influence of applying servant leadership in various organizational areas, as it significantly improves job satisfaction, perception of culture and commitment, and creativity (Hsiao et al., 2015; Harwiki, 2016; Neubert et al., 2016; Ozturk et al., 2021).

#### 1.2.3. Satisfaction with work resources

Satisfaction with work resources encompasses individual, leadership, group, and organizational levels. This allows for improvement in: (1) task-related resources (performance feedback), (2) implementation of new work resources (peer support), and (3) improvement of social resources in the workplace (supervisory coaching) (Schaufeli and Bakker, 2004; Spontón et al., 2019). Therefore, work resources include the physical, psychological, social, and organizational aspects of work, such as social support and job control, with three main objectives: (1) to help achieve work goals; (2) to reduce work demands and costs; and (3) to stimulate personal development, learning, and growth. In other words, work resources are important for coping with work demands and have intrinsic value (Schaufeli and Bakker, 2004).

In the educational sector, the applied leadership style influences not only productivity and profit outcomes but also the perception of teachers’ well-being and quality of life. However, it has not yet been explored in depth what factors in the work environment influence the relationship between leadership styles and life satisfaction, especially in the Peruvian context where most basic education teachers are forced to continue working with insufficient work resources and to experience an increase in emotional exhaustion, burnout, and low job satisfaction, these alterations can negatively affect teachers’ perception of their quality of life (Monsalve Mera et al., 2020; Celio, 2021; Guevara and Huyhua, 2021).

In Peru and Latin America, teacher’s life satisfaction is a relevant topic since it can affect their performance and retention in the profession. Satisfaction with job resources such as administrative support, training, and professional development can also play an important role in teacher’s life satisfaction (Nanjundeswaraswamy, 2021). With the aim of obtaining a better understanding, we seek to analyze satisfaction with job resources to evaluate the relationship between servant leadership style and life satisfaction of teachers, for a better understanding of the dynamics of the determinants of subjective well-being in the population of regular primary education teachers.

**Figure 1:**
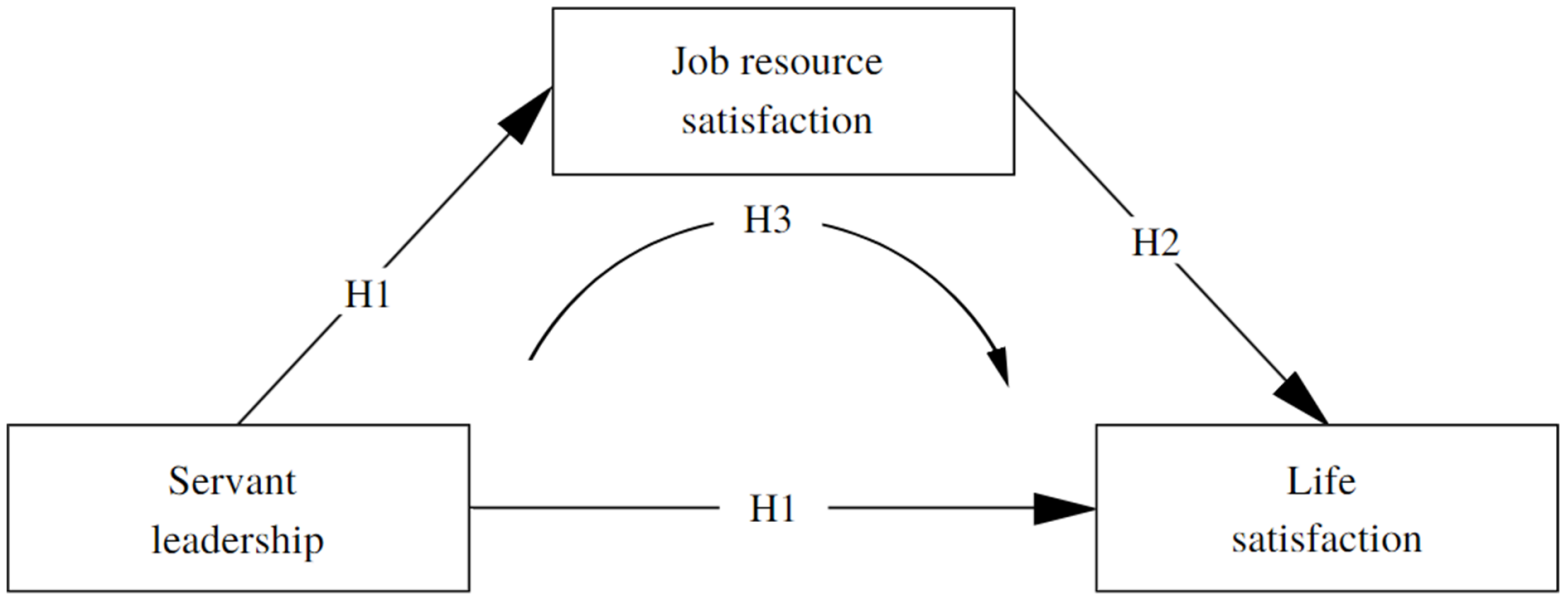
Theoretical model.

Based on the theoretical review, the following hypotheses are indicated (Figure 1):

*Hypothesis 1*: Servant leadership will have an effect on satisfaction with resources and satisfaction with life.

*Hypothesis 2*: Satisfaction with resources will have an effect on satisfaction with life.

*Hypothesis 3*: Satisfaction with resources will mediate the relationship between servant leadership and satisfaction with life.

## 2. Methods

### 2.1. Design and study population

A cross-sectional and explanatory study was designed considering latent variables represented by a system of structural equations (Ato et al., 2013). The effect size was analyzed considering the number of observed and latent variables in the model, the anticipated effect size (*λ* = 0.3), the desired statistical significance (*α* = 0.05), and the level of statistical power (1-*β* = 0.95), which considers a recommended minimum sample size of 207 (Soper, 2022). The data was obtained through a request for participation to the directors of four institutions. Prior to this, permission was sought from the corresponding educational authorities to conduct the research. After informing the participants about the anonymity of the research and obtaining their informed consent, the selection of participants was carried out. For participant selection, a convenience sampling was applied, choosing those who were available and willing to participate in the research. A total of 620 teachers participated, with ages ranging from 20 to 62 years (*M* = 35 and SD = 9.49). Most of the participants were female (63.1%), from the jungle region (44.8%), with a bachelor’s degree level of education (55.2%), and with a full-time contract (54.7%) (Table 1).

**Table 1:** Sociodemographic characteristics.

Characteristics		*n*	%
Gender	Female	391	63.1
	Male	229	36.9
Region of origin	Coast	230	37.1
	Jungle	278	44.8
	Sierra	112	18.1
Level of education	High school	192	31.0
	Doctorate	11	1.8
	Bachelor’s degree	342	55.2
	Master’s degree	75	12.1
Type of contract	Contract	195	31.5
	Full-time contract	339	54.7
	Part-time contract	86	13.9

### 2.2. Procedure

The research was approved by the Ethics Committee of a Peruvian University (2022-CEUPeU-026). Subsequently, during the period between July and August 2022, participants were invited to complete an online questionnaire through Google Forms. Prior to data collection, confidentiality rules and the principles of the Helsinki Declaration were followed, informing participants about the purpose of the research and obtaining their informed consent (Table 1).

### 2.3. Measures

Servant Leadership. The Spanish-adapted version (Rivera et al., 2017) of The Servant Leadership Short Scale (SLSS) was used to evaluate an important leadership model that prioritizes service to peers and subordinates as the main objective rather than seeking personal well-being or benefits for the organization, a cause, or a group of people. The scale consists of 14 items with 7 response options ranging from 1 (‘completely disagree’) to 7 (‘completely agree’). The internal consistency of the scale was good (*α* = 0.85).

Job Resource Satisfaction. The Spanish version of the Job Resources Satisfaction Questionnaire (CSRL_16) (Spontón et al., 2019) was used to evaluate the positive influence of job resources on workers’ well-being and performance. It has 16 items that respond to a five-category Likert scale: (1) completely disagree, (2) disagree, (3) neither disagree nor agree, (4) agree, and (5) completely agree. It also presents four dimensions with adequate internal consistency: Leader Resources (4 items, *α* = 0.91), Task Resources (4 items, *α* = 0.71), Team Resources (4 items, *α* = 0.88), and Organizational Resources (4 items, *α* = 0.81).

Life Satisfaction. The Spanish version of the Satisfaction with Life Scale (SWLS) (Calderón-De la Cruz et al., 2018) was used. It is a self-report measure that evaluates unidimensional life satisfaction with a total of five items scaled as an ordinal measure of five response options ranging from 1 = strongly disagree to 5 = strongly agree. The internal consistency was adequate, evaluated by McDonald’s Omega (*ω* = 0.90).

### 2.4. Data analysis

Initially, due to the fact that the questionnaires used belong to the category of self-administered and may lead to a common method bias, which refers to the measurement error that arises because of the specific method utilized in a study for the scale utilized, data collection techniques and analysis techniques (Podsakoff et al., 2003; Saris and Gallhofer, 2007). To correct this bias, there are both procedural and statistical solutions. In some cases, a research design that minimizes the effect of the common method bias can be used, such as using multiple sources of data or using different measures for the same construct. In other cases, statistical techniques can be applied to correct the common method bias. One statistical technique used in this study was the evaluation of the common method variance (CMV) through Harman’s single-factor test, which involves extracting a single factor and is expected to account for less than 50% of the total variance explained by the first factor in principal components analysis (Podsakoff et al., 2003).

Subsequently, descriptive statistics and correlations between study variables were calculated. Subsequently, the theoretical model of the study was analyzed using Structural Equation Modeling with the WLSMV (Weighted Least Squares Mean and Variance adjusted) estimator, used to analyze relationships between latent and observable variables. In these models, observed values can be biased or have measurement errors, affecting the accuracy of estimated parameters. WLSMV is used to mitigate this problem by providing a solution for estimating parameters in the presence of missing or biased data, as it is robust to inferential normality deviations (Little and Rubin, 2014). The fit evaluation was performed using the Comparative Fit Index (CFI), Tucker-Lewis Index (TLI), Root Mean Square Error of Approximation (RMSEA), and Standardized Root Mean Square Residual (SRMR). CFI and TLI values >0.90 (Bentler, 1990), RMSEA <0.080 (MacCallum et al., 1996), and SRMR <0.080 (Browne and Cudeck, 1992) were used. To analyze mediation in the model, the bootstrapping method with 5,000 iterations and a 95% confidence interval was employed (Yzerbyt et al., 2018). Regarding reliability analysis, the alpha (α) coefficient was used to evaluate the internal consistency of the scales used in the study (Figure 1).

The structural equation modeling analysis was conducted using R software version 4.0.5 and the *lavaan* package (66).

## 3. Results

### 3.1. Common method variance (CMV)

The results showed that the variance value was 38% through the Harman’s single factor test, which is below the threshold of 50% of variance explained (Table 2). Therefore, it was concluded that the dataset was free from common method bias (Podsakoff et al., 2003).

**Table 2:** Harman single factor test total variance explained.

Component	Initial eigenvalues			Extraction sums of squared loadings		
	Total	% var	Cumulative %	Total	% var	Cumulative %
1	13.41	0.38	0.38	13.41	0.38	0.38
2	5.54	0.16	0.54			
3	2.7	0.08	0.62			
4	1.32	0.04	0.66			
5	1.12	0.03	0.69			
6	1.04	0.03	0.72			
7	0.85	0.02	0.74			
8	0.72	0.02	0.76			
9	0.68	0.02	0.78			
10	0.61	0.02	0.8			
11	0.56	0.02	0.82			
12	0.51	0.01	0.83			
13	0.45	0.01	0.84			
14	0.43	0.01	0.86			
15	0.41	0.01	0.87			
16	0.4	0.01	0.88			
17	0.38	0.01	0.89			
18	0.34	0.01	0.9			
19	0.34	0.01	0.91			
20	0.3	0.01	0.92			
21	0.29	0.01	0.93			
22	0.27	0.01	0.93			
23	0.26	0.01	0.94			
24	0.24	0.01	0.95			
25	0.23	0.01	0.95			
26	0.22	0.01	0.96			
27	0.21	0.01	0.97			
28	0.2	0.01	0.97			
29	0.17	0.00	0.98			
30	0.16	0.00	0.98			
31	0.15	0.00	0.99			
32	0.14	0.00	0.99			
33	0.13	0.00	0.99			
34	0.12	0.00	1.00			
35	0.1	0.0	1.0			

### 3.2. Preliminary analysis

Descriptive statistics and correlations of the study variables are presented in Table 3. The analyses between the studied variables yielded highly significant correlation coefficients (*p* < 0.01). Bivariate analysis shows that service leadership is positively correlated with job resource satisfaction (*r* = 0.36, *p* < 0.01) and life satisfaction (*r* = 0.33, *p* < 0.01). Also, job resource satisfaction was positively correlated with life satisfaction (*r* = 0.48, *p* < 0.01).

**Table 3:** Descriptive statistics and correlations of the study variables.

Variable	*M*	SD	*α*	A	1	2	3
Servant leadership	70.34	12.25	0.96	−1.62	–		
Job resource satisfaction	48.43	6.89	0.93	−1.14	0.36**	–	
Life satisfaction	22.30	3.51	0.91	−2.25	0.33**	0.48**	–

SD, standard deviation; A, asymmetry; ** indicates *p* < 0.01.

### 3.3. Structural model

**Figure 2:**
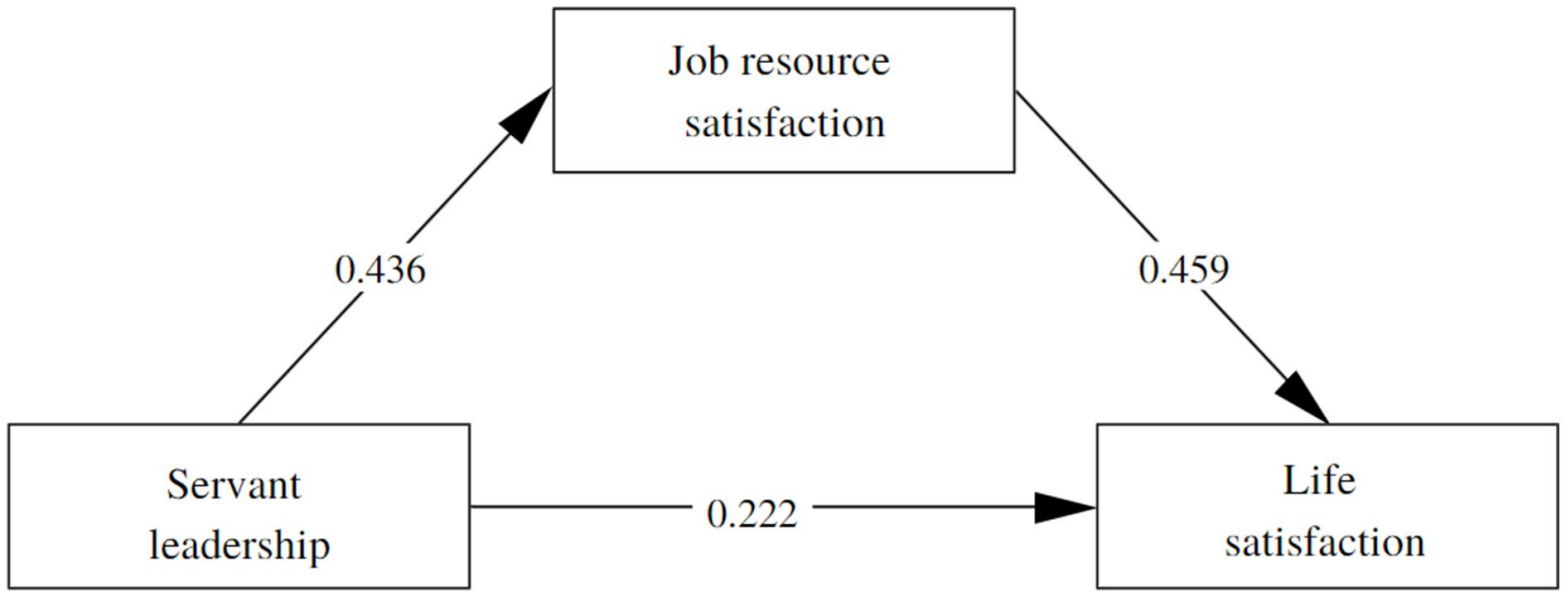
Results of the structural model.

In the analysis of the theoretical model, an adequate fit was obtained, *χ*^2^ = 2,658, df = 551, *p* < 0.001, CFI = 0.941, TLI = 0.936, RMSEA = 0.079, SRMR = 0.070 (Figure 2). The results confirm H1 regarding the positive influence of leadership on job resource satisfaction (*χ*^2^ = 0.436, *p* < 0.001) and life satisfaction (*χ*^2^ = 0.222, *p* < 0.001). Likewise, H2 was confirmed regarding the positive influence of job resource satisfaction on life satisfaction (*χ*^2^ = 0.436, *p* < 0.001).

### 3.4. Mediation model

For the mediation analysis, 5,000 iterations of bootstrapping were used. Thus, with respect to H3, the indirect effect is confirmed in which job resource satisfaction mediates the effect of servant leadership and life satisfaction, *β* = 0.100, *p* < 0.001, 95% CI [0.053, 0.161], and a total effect *β* = 0.224, *p* < 0.001, 95% CI [0.142, 0.318].

## 4. Discussion

This study evaluated the relationship between servant leadership and life satisfaction, considering the mediation of job resource satisfaction. This contributes to the literature on life satisfaction by investigating the effects of servant leadership on this critical indicator of happiness and well-being. Furthermore, the importance of human resource management in improving productivity and educational outcomes is understood (Firdaus et al., 2019). Therefore, servant leadership is critical for successfully leading and positively impacting organizations. Job resource satisfaction favors better performance, which fosters the achievement of work goals and more significant personal development and growth, which in turn contributes to life satisfaction (Table 3).

The results indicated that servant leadership had a positive effect on teachers’ life satisfaction. Previous studies support our findings, as servant leadership has been identified as an important indicator of happiness and well-being, finding that it is likely to play a critical role in increasing teachers’ life satisfaction (Chughtai, 2018; Li et al., 2018). This is because it promotes a positive and motivating work environment, as servant leadership has a person-centered attitude and tends to lead to stronger and more secure relationships in the school organization (van Dierendonck, 2010; Mittal and Dorfman, 2012; Eva et al., 2019). Servant leadership belongs to the holistic leadership approach, which seeks to develop capabilities based on altruistic and ethical orientations, prioritizing employees’ well-being and growth so that they are more committed and effective in their work (Eva et al., 2019). Therefore, teachers who are more satisfied with their lives create a conducive classroom environment for team success and the achievement of excellent work (Nagarajan et al., 2022). Our results extend the empirical evidence on the influence of servant leadership on life satisfaction. Other studies show that servant leadership did not have a significant impact on life satisfaction, which contradicts previous assumptions. However, this contradiction may be due to the presence of individual or collaborative work that weakens the direct effect of servant leadership on life satisfaction (Latif and Marimon, 2019; Pan et al., 2021).

The results indicated that job resource satisfaction has a positive effect on life satisfaction, as previously reported in which job resources predict employee well-being (Lyubomirsky et al., 2005; Salanova et al., 2012). This relationship is due to job resources being closely linked to employees’ work activity and being motivating due to their association with task characteristics such as clarity, autonomy, and feedback. This contributes to the construction of feelings of pride and enjoyment at work. Employees also interact with their peers, supervisors, and customers at work, which strengthens connections between employees and others. Therefore, the promotion of these resources through careful planning and implementation of activities and human resources contributes to the achievement of organizational goals (Schaufeli and Bakker, 2004; Salanova et al., 2012).

The results confirmed the mediating role of job resource satisfaction between servant leadership and life satisfaction. This is because leaders indirectly influence employee well-being by shaping their work environment and reinforcing their job resources (Chughtai, 2018). Therefore, the behavior of servant leaders would improve well-being by satisfying job resources. This relationship between job resources and life satisfaction is particularly relevant for teachers, as their work is crucial for the development and success of students as well as for society as a whole. It is concluded that servant leadership indirectly influences teacher well-being through the mediating mechanism of job resource satisfaction.

Under servant leadership, teachers can be ensured of having the resources they need to perform their job. This will allow them to have access to fundamental elements such as: (1) good communication and relationship with their superiors, clarification of information, constant feedback and recognition for their work; (2) intrinsic job characteristics that provide work challenges, sufficient time and resources to complete tasks, and the opportunity to use their skills; (3) a positive social work environment, with good relationships with their coworkers, and cooperation, coordination, productivity, and creativity in problem-solving; and (4) fair working conditions and appropriate organizational practices, including equitable salaries, non-monetary rewards, rest, opportunities for advancement, and continuous training (Spontón et al., 2019). This will make teachers feel satisfied and fulfilled with their work.

Teachers who lack adequate job resources are likely to experience a higher level of stress and workload, which can negatively affect their job and personal satisfaction (Chiniara and Bentein, 2016; Chughtai, 2018). Additionally, more resources will be needed to ensure teachers’ ability to provide quality education to their students, which in turn will adversely affect student performance and success.

### 4.1. Implications

This research expands the body of knowledge on the relationship between job resource satisfaction and servant leadership under the Job Demands-Resources (JD-R) theory. To improve quality of life, institutions must develop strategies to enhance job resources and improve organizational practices and resources. Additionally, it is essential to understand the importance of servant leadership for educational organizations. Therefore, it is crucial to implement interventions that foster servant leaders in organizations over time. This model can also be applied in other contexts, such as different countries and occupational sectors, using different qualitative and quantitative instruments to evaluate the organization as a whole.

### 4.2. Limitations

Regarding limitations, the cross-sectional design employed in this study precludes establishing causal relationships between variables. Therefore, it would be useful to conduct longitudinal research to evaluate the evolution of the relationships between variables over time. Likewise, the sample is composed mainly of teachers from Peru, which limits the generalizability of the results to other cultural and educational contexts. Thus, it is necessary to replicate the study in different regions and educational settings, in order to verify the consistency of the results in different situations. Although the scales used have demonstrated to be valid and reliable, there is always the possibility of bias in self-assessment. To obtain a more objective perspective, future research could include evaluations from multiple informants. The non-probabilistic sampling type and the online data collection may generate biases in the results, as some participants may not adequately complete the survey. In addition, only teachers with access to technology and who were interested in participating were included in the sample. To address this issue, it is important to replicate these results with data collected in other regions and through different sampling methods. Participants were assured that their responses would be anonymous and confidential, which helps reduce social desirability bias by allowing participants to feel more comfortable in responding truthfully.

## 5. Conclusion

In recent decades, leadership has become a research topic that has generated a lot of expectation, especially in developing countries that still need to improve the quality of teaching and learning experiences to inspire, communicate, and motivate students to achieve their academic goals. Through the mediation of job resource satisfaction, which includes physical, psychological, social, and organizational aspects, supported by servant leadership, job effort and the physiological and psychological costs associated with it are reduced, allowing for job goals to be achieved by stimulating personal growth and learning and having a significant impact on teacher well-being. The results confirm the mediation of job resource satisfaction between servant leadership and life satisfaction. Therefore, it is important for educational institutions to provide adequate resources to enhance job and life satisfaction for teachers.

## Data availability statement

The raw data supporting the conclusions of this article will be made available by the authors, without undue reservation.

## Ethics statement

The studies involving human participants were reviewed and approved by the Ethics Committee of the Universidad Peruana Unión (2022-CEUPeU-026). The patients/participants provided their written informed consent to participate in this study.

## Author contributions

RQ-D, WCM-G, and OM-B participated in the conceptualization of the idea. LP-Q, JS, and WCM-G were in charge of the methodology and software. WCM-G, RS-S, AAR-C, and JS performed validation, formal analysis, and research. WCM-G, AAR-C, and RQ-D were commissioned data curation and resources. LZS-S, WCM-G, RQ-D, AAR-C, and OM-B were carried out the writing of the first draft, review, editing, visualization, and supervision. All authors have read and approved the final version of the manuscript.
